# The Clarus Video System (Trachway) and direct laryngoscope for endotracheal intubation with cricoid pressure in simulated rapid sequence induction intubation: a prospective randomized controlled trial

**DOI:** 10.1186/s12871-019-0703-0

**Published:** 2019-03-04

**Authors:** Yen-Chu Lin, An-Hsun Cho, Jr-Rung Lin, Yung-Tai Chung

**Affiliations:** 1 Department of Anesthesiology, Chang Gung Memorial Hospital, No.5, Fuxing St., Guishan Dist., Taoyuan City, 333 Taiwan; 2 Clinical Informatics and Medical Statistics Research Center, Chang Gung University, Taoyuan City, Taiwan; 3 Graduate Institute of Clinical Medical Sciences (Joint Appointment), Chang Gung University, Taoyuan City, Taiwan; 4 College of Medicine, Chang Gung University, Taoyuan City, Taiwan

**Keywords:** Rapid sequence induction intubation, Cricoid pressure, The Clarus Video System, Direct laryngoscope

## Abstract

**Background:**

During an emergency endotracheal intubation, rapid sequence induction intubation (RSII) with cricoid pressure (CP) is frequently implemented to prevent aspiration pneumonia. We evaluated the CVS in endotracheal intubation in RSII with CP, in comparison with a direct laryngoscope (DL).

**Methods:**

One hundred fifty patients were randomly assigned to one of three groups: the CVS as a video stylet (CVS-V) group, the CVS as a lightwand (CVS-L) group and DL group. Primary outcomes were to assess the power of the CVS, compared with DL, regarding the first attempt success rate and intubation time in simulated RSII with CP. Secondary outcomes were to examine hemodynamic stress response and the incidence of complications.

**Results:**

The first attempt success rates within 30 s and within 60 s were higher in CVS-V and DL group than those in CVS-L group (*p* = 0.006 and 0.037, respectively). The intergroup difference for intubation success rate within 30 s was nonsignificant and almost all the patients were successfully intubated within 60 s (98% for CVS-L and DL group, 96% for CVS-L group). Kaplan-Meier estimator demonstrated the median intubation time was 10.6 s [95% CI, 7.5 to 13.7] in CVS-V group, 14.6 s [95% CI, 11.1 to 18.0] in CVS-L group and 16.5 s [95% CI, 15.7 to 17.3] in DL group (*p* = 0.023 by the log-rank test). However, the difference was nonsignificant after Sidak’s adjustment. The intergroup differences for hemodynamic stress response, sore throat and mucosa injury incidence were also nonsignificant.

**Conclusions:**

The CVS-D and DL provide a higher first attempt intubation success rate within 30 and 60 s in intubation with CP; the intubation time for the CVS-V was nonsignificantly shorter than that for the other two intubation methods. Almost all the patients can be successfully intubated with any of the three intubation methods within 60 s.

**Trial registration:**

ClinicalTrials.gov identifier: NCT03841890, registered on February 15, 2019 (retrospectively registered).

## Background

Although there has been no scientific evidence to prove that cricoid pressure (CP) will prevent aspiration pneumonia [1, 2], the majority of anesthesiologists (92%) in a national survey in the UK still use CP in rapid sequence induction intubation (RSII) [3]. Therefore, skillfully using a proper intubation device is obligatory for those anesthesiologists to ensure the successful endotracheal intubation with CP.

When properly applied, CP may not affect glottic view during endotracheal intubation with either a direct laryngoscope (DL) or a video laryngoscope [4–6]. However, the application of CP is likely to prolong the intubation time [5, 6]. Limited mouth opening or vulnerable teeth, which often accompany the patients requiring emergency intubation, are the two common factors to deter the intubators from using a laryngoscopic device. Besides, the blade of a laryngoscopic device is often too bulky for a narrow mouth opening, and the blade always bears a level force on upper incisors while the intubator is lifting epiglottis during intubation, which is liable to tooth fracture.

Intubating stylets with a slim handle have been proved to be superior to laryngoscopic devices for the endotracheal intubation in terms of ease of movement [7, 8] and prevention of dental injury [8]. The Clarus Video System (Trachway®, CVS) intubating stylet (Biotronic Instrument Enterprise Ltd., Tai-Chung, Taiwan), a video stylet, has been proved to be a comparable but faster solution to successful intubation than DL [9–11] in intubation without CP. Moreover, the CVS can also be operated as a lightwand when its red light is turned on. This alternative function is practically advantageous when the video image is blurred by mist, secretions or blood in the oral cavity or the intubators just lose their way in locating the glottis. The endotracheal tube will be initially guided into larynx in the dimly lit operating room by a bright glow moving in the anterior soft tissue of the neck and finally by the image of the trachea rings on the video screen. In addition, the CVS barely applies force on the teeth while an endotracheal intubation is being performed. As a result, dental trauma can be avoided.

How the CVS performs in endotracheal intubation with CP has not yet been evaluated in literature, so the study is the first to examine the capability of the CVS in intubation with CP. A lightwand (Surch-Lite; Aaron Medical, St Petersburg, FL), an intubation stylet without video assistance, was not recommended by Hodgson et al. as the first choice of endotracheal intubation with CP because of longer intubation time and higher failure rate for the first attempt [12]. Nevertheless, we hypothesized that the CVS could yield positive results in a study examining the capability of the CVS in endotracheal intubation with CP. In this prospective randomized study, we compare the use of the CVS and that of DL (Macintosh Laryngoscope) in patients undergoing endotracheal intubation in simulated RSII for the primary goals of the first attempt success rate and intubation time.

## Methods

This study was approved by Chang Gung Medical Foundation Institutional Review Board and a written informed consent was obtained from each patient. The patients participating in this clinical trial were older than 20 years of age and scheduled for elective surgery under general anesthesia. Patients were excluded if they had BMI (Body Mass Index) > 35 kg/m^2^, interincisor distance < 3 cm, poor dentition, upper airway tumor, limited neck mobility, pregnancy or history of difficult tracheal intubation.

One hundred fifty patients were enrolled and, based on computer-generated random numbers, were assigned to one of three groups: the CVS as a video stylet (CVS-V) group, the CVS as a lightwand (CVS-L) group and DL group. All intubations were executed by two anesthesiologists who are experienced in the use of the designated devices, Lin for CVS-V as well as DL group and Chung for CVS-L group. Both Chung and Lin have used the CVS in more than 200 cases, and Chung also has more than 10 years’ practice with lighted stylet [13].

After the intravenous line was checked and monitors, including electrocardiography, pulse oximetry and noninvasive blood pressure measurement, were correctively positioned, the patients breathed 100% oxygen for 3 min. Anesthesia was induced with fentanyl (2 μg/kg), lidocaine (20 mg), propofol (2 mg/kg) and rocuronium (1.2 mg/kg) intravenously. No assistant breathing was offered to all the patients before endotracheal intubation. Sixty seconds following the injection of rocuronium, each patient was intubated with the assigned device and an endotracheal tube of proper size while 30-40 N pressure was being applied on the cricoid cartilage by an assistant standing on a weighing scale [14]. Following checking the position and tapping of the tube, sevoflurane 4% in 50% oxygen with a fresh gas flow 4 L/min was initially provided from the circle system of the anesthesia machine and then the concentration of the inhalation anesthetic was adjusted in accordance with the patient’s need.

In this trial, successful intubation was defined as the intubation was completed within 30 s. Intubation time was counted from the inserting the device into the patient’s mouth to viewing the endotracheal tube into the trachea. The intubation time for patients who required more than one attempt was the sum of the times of all the intubation attempts. The following data were also collected: (1) airway parameters (Mallampati classification, thyromental distance, interincisor distance and neck circumference); (2) hemodynamic stress response; (3) sore throat and mucosa injury (documented by a blinded observer on the next day).

Statistical analyses were conducted using SPSS version 17.0 (SPSS Inc., Chicago, IL, U.S.) and SAS version 9.4 (SAS Institute Inc., Cary, NC, U.S.). The study was designed to allow the intergroup difference of 20% for first attempt success rate to detect at 5% level of significance with a power of 80%. Categorical data were analyzed by the chi-square test and continuous data by one-way analysis of variance. Hemodynamic changes responsive to endotracheal intubation were tested by analysis of covariance, using preinduction variables as the covariates. Bonferroni post hoc tests were performed where appropriate. The log-rank test with Sidak correction was used to compare the intergroup difference for the Kaplan-Meier curves that were obtained from the time to successful intubation with one attempt for each patient. A *p* value less than or equal to 0.05 was considered to be statistically significant.

## Results

**Fig. 1:**
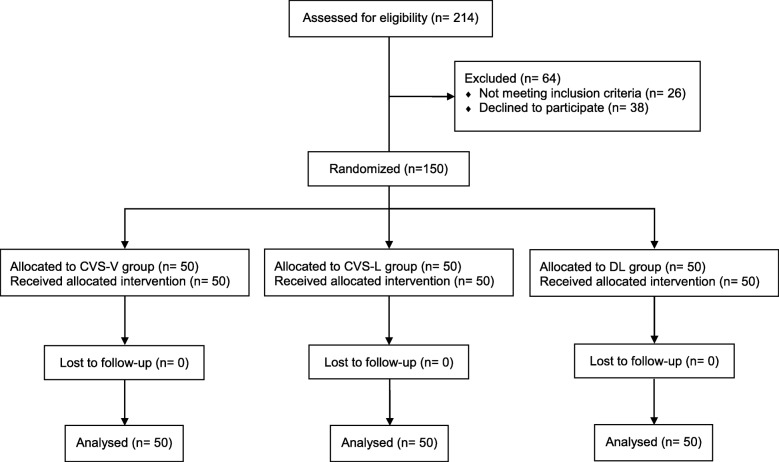
CONSORT flowchart

A total of 214 patients undergoing elective surgery were screened between November 2016 and April 2018, of which 64 were excluded because they refused to participate or did not meet one or more of the inclusion criteria. The others were randomly allocated into three groups (Fig. 1). Demographics and airway characteristics of the patients in three groups were similar (Table 1).

**Table 1:** Demographic data, airway characteristics, complications and hemodynamic responses

	CVS-V group (*n* = 50)	CVS-L group (*n* = 50)	DL group (*n* = 50)	*p* value
Demographic data				
Age (years)	46 ± 12	47 ± 14	48 ± 12	0.575
ASA class (I/II/III)	16/31/3	15/33/2	10/36/4	0.634
Gender (M/F)	23/27	29/21	24/26	0.437
Body height (cm)	163 ± 8	165 ± 8	163 ± 8	0.313
Body weight (kg)	66 ± 11	68 ± 12	65 ± 13	0.478
BMI (kg/m^2^)	25 ± 3	25 ± 3	24 ± 4	0.878
Airway characteristics				
Mallampati classification (1/2/3/4)	8/16/20/6	12/17/12/9	12/11/19/8	0.512
Thyromental distance (cm)	6.5 ± 0.7	6.4 ± 0.7	6.6 ± 0.8	0.360
Interincisor distance (cm)	4.5 ± 0.8	4.6 ± 0.6	4.7 ± 0.6	0.304
Neck circumference (cm)	38 ± 5	39 ± 5	38 ± 4	0.431
Complications				
Sore throat in the next day^a^ (n; none/mild/moderate/severe)	37/10/2/1	34/11/5/0	28/17/4/1	0.445
Mucosa injury	2	1	0	0.360
Hemodynamic response^b^				
Patients successfully intubated at the first attempt	49	44	49	
Mean arterial pressure (mmHg)				
Preinduction	98 ± 12	101 ± 15	98 ± 12	0.321
1 min after intubation	98 ± 23^c^	107 ± 21	104 ± 22	0.164
5 min after intubation	74 ± 14	78 ± 16	77 ± 17	0.575
Heart rate (bpm)				
Preinduction	74 ± 14	77 ± 12	70 ± 11	0.149
1 min after intubation	89 ± 15	94 ± 17	88 ± 14	0.571
5 min after intubation	84 ± 15	86 ± 16	81 ± 14	0.926

Values are shown as mean ± standard deviation or number

^a^ Sore throat was graded according to numerical rating scale (NRS): none, NRS = 0; mild, NRS = 1–3; moderate, NRS = 4–6; severe, NRS = 7–10

^b^ Analysis of hemodynamic response excluded patients who need second attempt. Preinduction variables are referred to as covariate of 1 min or 5 min after intubation variables in the analysis of covariance (ANCOVA). No significant hemodynamic response to endotracheal intubation was seen in any of the three groups

^c^ No statistical difference versus preinduction value within the group

The first attempt success rate (in either within 30 s or within 60 s) of CVS-V and DL group was significantly higher than that of CVS-L group (*p* = 0.006 and 0.037, respectively) (Table 2). Forty-seven patients (94%) in CVS-V group, 47 in DL group (94%) and 38 in CVS-L group (76%) had their intubation completed within 30 s at the first attempt. Of the ten patients requiring time between 30 and 60 s to be intubated at the first attempt, 2 were from CVS-V group, 6 from CVS-L group and 2 from DL group, respectively. Eight patients required two attempts to be intubated, 1 patient in CVS-V group by the CVS-V, 6 in CVS-L group by the CVS-L and 1 in DL group by the CVS-V (due to unseen glottis with DL at the first attempt). However, the intergroup difference for intubation success rate (including patients who had 2 attempts of intubation) within 30 s was nonsignificant (94% for CVS-L and DL group, 82% for CVS-L group). Almost all the patients were intubated within 60 s (98% for CVS-L and DL group, 96% for CVS-L group).

**Table 2:** Data of endotracheal intubations

	CVS-V group (*n* = 50)	CVS-L group (*n* = 50)	DL group (*n* = 50)	*p* value
Patients successfully intubated				
At the first attempt within 30 s	47 (94)	38 (76)	47 (94)	0.006
At the first attempt within 60 s^a^	49 (98)	44 (88)	49 (98)	0.037
Success within 30 s (including two attempts)	47 (94)	41 (82)	47 (94)	0.069
Success within 60 s (including two attempts)	49 (98)	48 (96)	49 (98)	0.773
Median time to successful intubation at the first attempt (s)	10.6 (7.5 to 13.7)	14.6 (11.1 to 18.0)	16.5 (15.7 to 17.3)	0.023^b^

Values are shown as number (%) or median (95% CI)

^a^ Intubation tools at the second attempt were the same in both CVS-V and CVS-L group. In the DL group, CVS-V was used in this case due to unseen glottis at the first attempt

^b^ Data from Kaplan-Meier estimator with *p* value of log-rank test. However, after Sidak’s adjustment for multiple comparisons for the log-rank test, the *p* values were all more than 0.05 in three comparisons (*p* = 0.0566 between CVS-V group and CVS-L group, *p* = 0.0762 between CVS-V group and DL group, and *p* = 0.9998 between CVS-V group and CVS-L group)

**Fig. 2:**
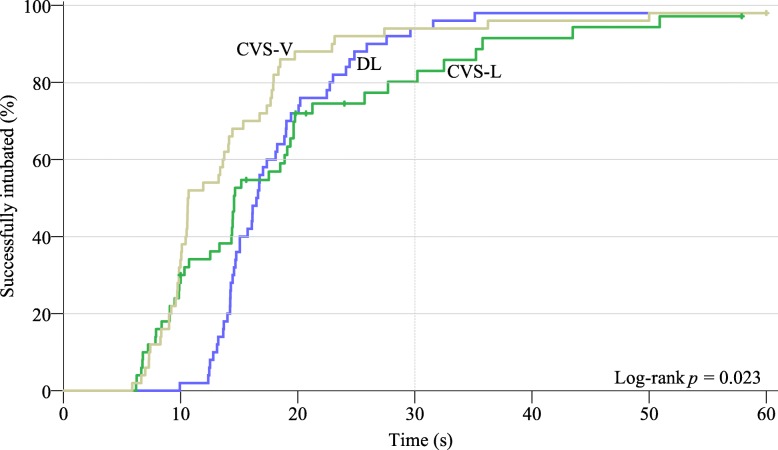
Duration of the successful intubation at the first attempt demonstrated by Kaplan-Meier estimator. Vertical ticks mark the time point when the first intubation attempt failed (censored observation). Kaplan-Meier estimator demonstrates that the intubation time in CVS-V group is consistently shorter than that in the other two groups. And the intubation time in CVS-L is the most inconsistent. The *p* value was 0.023 by the log-rank test. However, after Sidak’s adjustment for multiple comparisons for the log-rank test, the *p* values were more than 0.05 between the groups

If the patients with failed intubation at the first attempt were treated as censored observations, Kaplan-Meier estimator (Fig. 2) demonstrates that more patients in CVS-V group than in the other two groups have their intubation completed in shorter time. The median time of successful intubation at the first attempt was faster in CVS-V (10.6 s [95% CI, 7.5 to 13.7]) than in CVS-L group (14.6 s [95% CI, 11.1 to 18.0]) or in DL group (16.5 s [95% CI, 15.7 to 17.3]) (Table 2). The *p* value was 0.023 by the log-rank test, but the intergroup difference was nonsignificant after Sidak’s adjustment (*p* = 0.0566 between CVS-V group and CVS-L group, *p* = 0.0762 between CVS-V group and DL group, and *p* = 0.9998 between CVS-V group and CVS-L group).

Although mean blood pressures in CVS-V group appeared the least responsive to intubation, the intergroup difference was nonsignificant (Table 1). There was no patient who had oxygen saturation below 90% during intubation. Sore throat and mucosa injury occurred with a similar frequency in the three groups (Table 1).

## Discussion

In a large randomized clinical trial (3472 cases) conducted in urban academic centers, intubation with CP failed to show an advantage over intubation without CP in RSII in terms of preventing pulmonary aspiration [2]. However, the authors also mentioned that the results of the study may not be applied to emergency cases outside operating rooms where there are supposed to be more manpower and equipment, and patients probably has more adequate muscle relaxation for endotracheal intubation. Aspiration pneumonia is still a major concern to many anesthesiologists, so they will not hesitate to apply CP while intubating patients with risks of the complication [3].

As compared to DL, the video laryngoscopes during endotracheal intubation are associated with less neck manipulation, a better glottic view and a higher success rate of intubation in normal or difficult airways [15], but they don’t usually guarantee shorter intubation time [16, 17]. Moreover, like DL with a bulky blade, they are not usually chosen for patients presenting with limited mouth opening or fragile incisors. On the contrary, the CVS can be a tool of choice in such patients thanks to its slim stylet and video screen. The CVS has also been proved to provide faster endotracheal intubation than DL [10] and the Airway Scope (Pentax, Tokyo, Japan) in a simulated difficult airway [18]. Therefore, we assumed that the CVS is a preferable tool of intubation over laryngoscopic devices in intubation with CP.

Regarding the learning curve of the CVS, an inexperienced trainee can be proficient in using it after a few practices. Ten times of practice is sufficient for the inexperienced to learn the proper use of the CVS and after practicing on 20 patients, they are likely to accomplish intubation with the CVS at the first attempt in a mean intubation time less than 20 s [19]. As compared with the studies of intubation with the CVS without CP [10, 11], the median intubation time and success rate at the first attempt of intubation for CVS-V group in our study (50 cases) is 10.6 s [95% CI, 7.5 to 13.7] and 94%, 15 s [IQR, 12 to 19] and 89.9% in Yang et al’s study (200 cases) [10] and 9 s (mean) [SD, 4] seconds and 100% in Hsu et al’s study (30 cases) [11] (all the data calculated based on same definition of intubation time). Thus, CP does not appear to significantly affect the intubation time in CVS-V group in our study. When it comes to endotracheal intubation with CP, the intubation time in any of the three groups of our study is much shorter than that (78.8 s [SD, 41.2]) in the study by Hodgson et al. [12]. Therefore, with video assistance, the CVS-V as a video stylet is a handy device for endotracheal intubation with CP.

During intubation in CVS-L group, the application of CP may displace the larynx and cause difficulty for the operator to move the tube into the larynx, and under downward direction of the force the esophagus gets closer to anterior neck skin, so the false positive transillumination on the anterior neck tissue becomes more frequent. Nevertheless, the intubation still can be facilitated by checking the position of the tube on the video screen. Endotracheal intubation with the CVS-L may not be as straightforward as that with the CVS-V, but it can be accomplished sooner than that with a lightwand per se [12].

This study was conducted in simulated RSII while patients’ muscle power was not being monitored during anesthesia. Instead, we provided rocuronium 1.2 mg/Kg, which is proved by Magolian et al. to allow onset time (55 ± 14 s) [20]. The patients were intubated 1 min after injection of rocuronium and all the intubation conditions in the study were acceptable.

Thirty seconds was set as a cutoff point for the successful intubation time based on the research team’s experience and literature [7, 10, 11] where an intubation is usually completed in less than 30 s with either DL or the CVS. This study showed that the median time to successful intubation is within expected 30 s in all of the three groups.

There are three limitations in the study. Firstly, Lin did all the intubations in both CVS-V and DL group, so personal bias was possibly involved in the results. However, the results regarding our primary goals do not deviate from those in previous studies where the intubation using the CVS without CP [10, 11], so the personal bias should be minimal. Secondly, this was a randomized controlled study about how the CVS and DL perform in endotracheal intubation with CP, so ethically we need to conduct a study on patients whose airway conditions meet the indications to the use of the CVS and DL alike. Thirdly, the results revealed that the intubation time in CVS-V group was shorter than those in the other groups, but the intergroup difference was nonsignificant. It seems that the intergroup difference for intubation time is less than we expected and we should have had a larger sample size of patients to prove our hypothesis. Nevertheless, it is still worthwhile to further study how powerful the CVS-V can be in intubation in RSII when patients present with limited mouth opening or fragile incisors, which are two specific indications where an intubation stylet may be more advantageous over a laryngoscopic device.

## Conclusions

Both of the CVS-V and DL can provide comparably higher first attempt intubation success rate within 30 s as well as within 60 s in endotracheal intubation with CP. Intubation time with the CVS-V was nonsignificantly shorter than that with the other two intubation methods. Almost all the patients can be successfully intubated with any of the three intubation methods within 60 s.

## Abbreviations

ANCOVA: Analysis of covariance
ASA: American Society of Anesthesiologists
AWS: Pentax airway scope
BMI: Body mass index
CVS: The Clarus Video System
CVS-L: The Clarus Video System as a lightwand
CVS-V: The Clarus Video System as a video stylet
DL: Direct laryngoscope
NRS: Numerical rating scale
RSII: Rapid sequence induction intubation
